# Long noncoding RNA TLNC1 promotes the growth and metastasis of liver cancer via inhibition of p53 signaling

**DOI:** 10.1186/s12943-022-01578-w

**Published:** 2022-04-27

**Authors:** Kefei Yuan, Jiang Lan, Lin Xu, Xuping Feng, Haotian Liao, Kunlin Xie, Hong Wu, Yong Zeng

**Affiliations:** 1 Department of Liver Surgery & Liver Transplantation, State Key Laboratory of Biotherapy and Cancer Center, West China Hospital, Sichuan University and Collaborative Innovation Center of Biotherapy, Chengdu, 610041 China; 2 Laboratory of Liver Surgery, West China Hospital, Sichuan University, Chengdu, 610041 China

**Keywords:** lncRNA, TLNC1, TPR, p53, Liver cancer

## Abstract

**Background:**

Long non-coding RNAs (lncRNAs) have been demonstrated to play vital roles in cancer development and progression. However, their biological roles and function mechanisms in liver cancer remain largely unknown.

**Methods:**

RNA-seq was performed with clinical hepatoma tissues and paired adjacent normal liver tissues to identify differentially expressed lncRNAs. qPCR was utilized to examine the expression levels of lncRNAs. We studied the function of TLNC1 in cell growth and metastasis of hepatoma with both cell and mouse models. RNA-seq, RNA pull-down coupled with mass spectrometry, RNA immunoprecipitation, dual luciferase reporter assay, and surface plasmon resonance analysis were used to analyze the functional mechanism of TLNC1.

**Results:**

Based on the intersection of our own RNA-seq, TCGA RNA-seq, and TCGA survival analysis data, TLNC1 was identified as a potential tumorigenic lncRNA of liver cancer. TLNC1 significantly enhanced the growth and metastasis of hepatoma cells both in vitro and in vivo. TLNC1 exerted its tumorigenic function through interaction with TPR and inducing the TPR-mediated transportation of p53 from nucleus to cytoplasm, thus repressing the transcription of p53 target genes and finally contributing to the progression of liver cancer.

**Conclusions:**

TLNC1 is a promising prognostic factor of liver cancer, and the TLNC1-TPR-p53 axis can serve as a potential therapeutic target for hepatoma treatment.

**Supplementary Information:**

The online version contains supplementary material available at 10.1186/s12943-022-01578-w.

## Background

Liver cancer is the sixth most common malignancies and the third leading cause of cancer death worldwide [1]. The high mortality of liver cancer has been recognized to be directly caused by metastasis and postoperative recurrence [2]. However, at present, very limited approaches can be utilized in clinic to prevent the recurrence and metastasis of liver cancer [3]. In order to treat the recurrence and metastasis of liver cancer more effectively, it is of great importance to investigate the molecular mechanisms of the recurrence and metastasis of liver cancer and identify potential therapeutic targets.

Long non-coding RNAs (lncRNAs) belong to a class of non-coding RNAs (ncRNAs) that are > 200 bps [4]. Recently, lncRNAs have been demonstrated to play vital roles in the development and progression of many different cancer types, including breast cancer, lung cancer and liver cancer [5–7]. To exert the regulatory functions, lncRNAs can serve as a sponge for messenger RNAs (mRNAs) and microRNAs (miRNAs). For example, lncRNA TINCR controls the somatic tissue differentiation through interaction with a range of differentiation mRNAs [8]. On the other hand, although named as “non-coding RNAs”, a few lncRNAs have been shown to encode peptides. For instance, lncRNA HOXB-AS3 has been demonstrated to encode a peptide that is able to suppress PKM splicing and subsequent metabolic reprogramming, finally repress colon cancer growth [9]. Last but not least, because of the relatively large size that renders lncRNAs with more complex structures, many lncRNAs show the potential to interact with proteins and regulate the protein functions directly or the protein-protein interactions. As an example of lncRNAs with this kind of function, lncRNA HOTAIR serves as a scaffold for two distinct histone modification complexes, polycomb repressive complex 2 (PRC2) and LSD1/CoREST/REST complex. HOTAIR tethers two distinct complexes to enable RNA-mediated assembly of PRC2 and LSD1 and coordinate targeting of PRC2 and LSD1 to chromatin for coupled histone H3 lysine 27 methylation and lysine 4 demethylation [10]. Although the functions and mechanisms of many lncRNAs have been revealed, the roles for most of the lncRNAs remain unknown.

As one of the most famous tumor suppressors, p53 has been demonstrated to be frequently inactivated in liver cancer [11], suggesting that p53 plays a critical role in the development and progression of liver cancer. In cells where p53 is not inactivated, this protein has to translocate from the cytoplasm into the nucleus in order to exert its function as a transcription factor [12]. The nuclear translocation of p53 is a dynamic process, which means the nuclear p53 can also be translocated back into the cytoplasm. The nuclear export process of p53 depends on chromosome maintenance region 1 (CRM1), which is also known as exportin 1 [13]. Recently, translocated promoter region (TPR), a component of the nuclear pore complex (NPC), has been identified as another member of the nuclear export complex of p53 [14]. Mechanistically, TPR has been shown to interact with CRM1 [15], which then mediate the CRM1-dependent nuclear export of p53. Consistently, TPR can be co-immunoprecipitated by anti-p53 antibody, suggesting that TPR may interact with p53 [14]. However, whether the interaction between TPR and p53 is direct or mediated by another component, such as CRM1, remains to be investigated.

In this study, we identified an oncogenic lncRNA, termed as TLNC1 (tumorigenic long noncoding RNA on chromosome 1p13), that was highly expressed in liver cancer tissues. TLNC1 was previously known as linc01134, which has been demonstrated to promote the progression and metastasis, as well as confer oxaliplatin resistance in hepatocellular carcinoma (HCC) by several recent studies [16–19], indicating that this lncRNA plays vital roles in HCC. However, the biological functions and molecular mechanisms of TLNC1 require to be further investigated. In our study, we showed that overexpression of TLNC1 promotes the growth and metastasis of liver cancer cells both in vitro and in vivo. Moreover, we revealed that TLNC1 exerted its tumorigenic function through interaction with TPR and facilitating the interaction between TPR and CRM1, which finally induced the nuclear export of p53 and promoted the progression of liver cancer.

## Materials and methods

### Cell lines

Hep3B, HCCLM3, SK-Hep1 and 293 T cells were obtained from the Cell Bank Type Culture Collection of the Chinese Academy of Sciences, Shanghai, China. SNU-449 cells were purchased from ATCC (Manassas, VA, USA). Hep3B cells were cultured in Minimum Essential Medium (HyClone, Logan, UT, USA) containing 10% FBS (HyClone), 1% penicillin/streptomycin (HyClone) at 37 °C and 5% CO_2_. All other cells were cultivated in Dulbecco’s modified Eagle’s medium (HyClone, Logan, UT, USA) containing 10% FBS (HyClone), 1% penicillin/streptomycin at 37 °C and 5% CO_2_.

### Clinical samples

A total of 83 liver cancer tissues and corresponding adjacent normal tissues were collected from deidentified liver cancer patients undergoing hepatectomy at West China Hospital (WCH; Chengdu, China). The protocols used in this study were approved by the Ethical Review Committees of Sichuan University, and written informed consent was obtained from all patients.

### In vivo tumor growth and metastasis models

The animal studies were authorized by the Animal Ethic Review Committees of the West China Hospital. For the xenograft tumor model, 5 × 10^6^ cells were injected subcutaneously into nude mice (BALB/c, 6 weeks of age, *n* = 5 per group) (Vital River Laboratories, Beijing, China). After 4 weeks, the mice were euthanized and tumors were excised, pictured and weighed. Tumor volume was calculated as length × width^2^ × 0.5. For the orthotopic implanted tumor model, 2 × 10^6^ cells were injected into the livers of mice. After 4 weeks, the tumor growth in the livers of mice was visualized using the IVIS@ Lumina II system (Caliper Life Sciences, Hopkinton, MA) 15 min after intraperitoneal injection of 3 mg of D-Luciferin (ABP Biosicences, USA) in 200 μl of sterile PBS without magnesium or calcium. Then, the mice were euthanized and livers were excised and pictured. The number of tumor nodules was counted in H&E staining in tissue sections of livers under the AX10 imager A2/AX10 cam HRC microscope (Zeiss, Oberkochen, Germany). For the lung metastasis model, 2 × 10^6^ Hep3B, HCCLM3 cells and 1 × 10^6^ SK-Hep1 cells were inoculated through tail vein into the nude mice. After 1 week, another injection with the same cell amount was performed to each pre-injected mouse. Then, after another 4 weeks, the tumor growth in the lungs of mice was visualized using the IVIS@ Lumina II system. After that, the mice were sacrificed and lungs were dissected and pictured. The number of pulmonary metastatic nodules was counted in H&E staining in tissue sections of lungs. All animal experiments were strictly implemented in compliance with the NIH Guide for the Care and Use of Laboratory Animals.

### RNA sequencing

The detailed procedures were described previously [2]. In brief, total RNA extracts of liver cancer and paired peritumoral normal tissues (*n* = 11) were used for RNA-seq to identify the differentially expressed lncRNAs. Differential expression analysis between two groups was performed with DESeq R package. To control the False Discovery Rate (FDR), the *p* values were adjusted with the Benjamini-Hochberg method. The cutoff value of differentially expressed lncRNAs was set as |log2[fold change (FC)]| > 1 and FDR < 0.05. To illustrate the downstream target of TLNC1, RNA-seq was performed with shNC and shTLNC1 HCCLM3 cells (*n* = 3).

### TCGA data analysis

RNA-sequencing data and clinical information of liver cancer samples (*n* = 370), as well as RNA-sequencing data of 50 adjacent non-cancerous samples were obtained from cBioPortal (www.cbioportal.org). X-tile plots (version 3.6.1, Yale University School of Medicine) were used to select the optimum cutoff for the expression of overlapping between differentially expressed lncRNAs of WCH and TCGA cohorts based on the association of lncRNA levels with the patients’ OS and DFS. At a certain cutoff value, the lncRNAs with survival analysis by X-tile plots showing OS (*p* < 0.05) and DFS (p < 0.05) were identified.

### RNA extraction and real-time quantitative PCR

Total RNA was extracted using Trizol (Invitrogen, USA). cDNA was prepared using HiScript III All-in-one RT SuperMix Perfect for qPCR (Vazyme, Nanjing, China). Real-time PCR was performed using ChamQ SYBR Color qPCR Master Mix (Vazyme, Nanjing, China) according to the manufacturer’s instructions. The primer sequences were listed in Table S1.

### Constructs and transfection

The full-length and truncated TPR plasmids (Flag-TPR) were obtained from GeneChem (Shanghai, China). The plasmids were transfected into 293 T cells with GenJet™ Plus reagent (SignaGen Laboratories, Maryland, USA). Specific small interfering RNA (siRNA) targeting TPR was purchased from Santa Cruz (sc-45,343). The transient transfection of siRNA was performed using Genmute™ Reagent (SignaGen Laboratories, Maryland, USA) according to the manufacturer’s instructions.

### Construction of stable cell lines

For stable overexpression of TLNC1, the human TLNC1 sequence (NCBI Reference Sequence: NR_024455.1) was synthesized and cloned into the ORF lentiviral expression vector pEZ-Lv217 (GeneCopoeia, Rockville, USA), named as LV-TLNC1. For stable knockdown of TLNC1, specific cDNA oligonucleotides targeting TLNC1 were cloned into the shRNA expression plasmid psi-LVRU6MP (GeneCopoeia, Rockville, USA). The target sequences were as follows: TLNC1 shRNA-1 GGACAGGTTTGAGCTAGAA; TLNC1 shRNA-2 CCACTCCCAGCTTGTTCAT. For construction of stable transfection cell lines, the lentiviral particles were used to transfect indicated cells according to the manufacturer’s instructions.

### RNA fluorescence in situ hybridization (FISH)

LncRNA FISH was performed to determine the subcellular location of TLNC1. FISH probes against TLNC1 were designed and synthesized by RiboBio (RiboBio Biotechnology, Guangzhou, China). FISH was conducted using the Fluorescent In Situ Hybridization Kit (RiboBio Biotechnology, Guangzhou, China), according to the manufacturer’s instructions. Fluorescent images were captured by the A1R + MP confocal laser microscope system (Nikon, Tokyo, Japan).

### Cell counting Kit-8 (CCK-8) cell viability assay

Cells were suspended with 100 μl medium, seeded into the 96-well plate (2000 cells/well), and cultured for indicated times. 10 μl CCK-8 (Beyotime Biotechnology, Shanghai, China) was added into each well, followed by incubation in cell incubator for 2 h. The absorbance was read at 450 nm by the Eon™ Microplate Reader (BioTek, Whiting, VT, USA).

### EdU cell proliferation assay

Cells were suspended with 500 μl medium, seeded into the 24-well plate (2 × 10^4^ cells/well), and cultured until the cells reached the proper growth status. After discarding the original culture medium, 300 μl medium containing 5-Ethynyl-2′-deoxyuridine (EdU) (50 μM) (RiboBio Biotechnology, Guangzhou, China) was added to each well and incubated in cell incubator for 2 h. Discard the EdU medium and rinse the cells with PBS for 2 times (5 min/time). After that, the cells were fixed with 4% paraformaldehyde in PBS for 30 min at room temperature, quenched in glycine solution (2 mg/mL) and permeabilized with 0.5% Triton X-100 for 10 min, followed by staining with Apollo dye solution for 30 min. Images were captured using the OBSERVER D1/AX10 cam HRC microscope (Zeiss, Oberkochen, Germany) with a charge-coupled device (CCD) camera.

### Wound-healing migration assay

For wound-healing migration assay, confluent monolayer cells were wounded with a p20 pipette tip. The images were taken after PBS wash (time 0 h). The cells were cultured in standard medium. After 1 day, the images were taken again (time 24 h). Three separate fields were photographed for each plate (one representative image was shown in the figure).

### Transwell migration and Matrigel invasion assays

Transwell membrane (8 μm pore size, 6.5 mm diameter) (Corning Costar, Corning, NY, USA) was used for both assays. For transwell migration assay, 2.5 × 10^4^ cells were plated in the top chambers. Serum-free medium was added into the top chambers and migration-inducing medium (with 10% FBS) was added into the bottom chambers. After 24 h, the filters were fixed with 4% paraformaldehyde. Cotton swabs were used to scrape the cells on the upper side of the membrane and crystal violet was used to stain the cells on the bottom side of the membrane. The membranes were washed with PBS and photographed after dry out. For Matrigel invasion assay, the top chambers were coated with Matrigel before 5 × 10^4^ cells were plated. Images were taken after 24 h.

### RNA pull-down assay

TLNC1 was transcribed in vitro with Ribo™ RNAmax-T7 Biotin-labeled Transcription Kit according to the manufacturer’s instructions (RiboBio Biotechnology, Guangzhou, China). The biotin-labeled TLNC1 was then pulled down by Pierce™ Streptavidin Magnetic Beads (Thermo Scientific) and incubated with protein lysates of HCCLM3 cells. Finally, the precipitated proteins were identified by mass spectrometry (LTQ Orbitrap Velos Pro, Thermo Scientific).

### Immunoblot analysis

RIPA buffer (Beyotime Biotechnology, Shanghai, China) was used for cell lysis and BCA protein assay kit (Beyotime Biotechnology, Shanghai, China) was used for quantification of protein concentrations. Primary antibodies were from Santa Cruz (β-actin, 1:1000, mouse monoclonal, sc-69,879), Zen Bioscience (GAPDH, 1:1000, mouse monoclonal, 200,306), Abcam (TPR, 1:1000, rabbit polyclonal, ab229352; CRM1, 1:1000, rabbit monoclonal, ab191081), Cell Signaling Technology (p53, 1:1000, Rabbit Polyclonal, 2527; PARP, 1:1000, Rabbit Polyclonal, 9542; Flag tag, 1:1000, Rabbit Polyclonal, 14,793). Secondary antibodies were from Santa Cruz (Goat anti-mouse IgG-HRP, 1:5000, sc-2005; Goat anti-rabbit IgG-HRP, 1:5000, sc-2004). For preparation of cytoplasmic and nuclear proteins, NE-PER nuclear and cytoplasmic extraction reagents (Pierce, Thermo Scientific) were used according to the manufacturer’s instructions. Immunoprecipitation was performed using Pierce™ Crosslink Magnetic IP/Co-IP kit (Thermo Scientific) according to the manufacturer’s instructions.

### RNA immunoprecipitation (RIP)

The Magna RIP™ RNA-binding Protein Immunoprecipitation Kit (Millipore, Massachusetts, USA) was utilized to perform RIP assay following the manufacturer’s protocol. Briefly, 5 μg of specific antibody or normal IgG (Millipore, Massachusetts, USA) were first linked to magnetic beads, and then incubated with cell lysates overnight at 4 °C. Proteinase K digestion buffer was used to treat the washed RNA-protein complexes. Phenol: chloroform: isoamyl alcohol was used to purify the coprecipitated RNAs. Subsequently, the purified RNA samples were analyzed by qPCR to assess the enrichment of TLNC1 to TPR.

### Dual luciferase reporter assay

The indicated SK-Hep1 and HCCLM3 cells were plated in 24-well plates at 2 × 10^4^ cells per well 24 h before transfection of luciferase reporter plasmids. PG13-luc luciferase reporter [20] and pRL-CMV plasmid (Renilla luciferase, Promega) were transfected together into indicated hepatoma cells. After 48 h, luciferase activity was determined by a dual luciferase reporter assay system (Promega) according to the instructions of the manufacturer.

### Immunofluorescence analysis

Cells were cultured on coverslips in a 24-well plate (MatTek, Ashland, MA). For immunostaining, the cells were fixed in 4% paraformaldehyde, permeabilized with 0.2% Triton X-100, and blocked with 5% BSA for 1 h. After that, cells were incubated with primary antibodies (100 times dilution) for 1 h and washed three times with PBST (PBS with 0.1% Tween-20). After incubation with appropriate fluorophore-conjugated secondary antibodies (Molecular Probes) and DAPI (Thermo Fisher), the coverslips were mounted on slides. The pictures were photographed with AX10 imager A2 microscope (Carl Zeiss MicroImaging).

### Recombinant protein expression and purification

His-TPR, His-CRM1 and His-p53 recombinant proteins were expressed in HEK293T cells and purified with Ni beads (17–5318-06, GE Healthcare). Experiments with recombinant proteins were performed in the presence of a synthetic peptide comprising the nuclear export signal (NES) derived from PKI (ELALKLAGLDIN) to strengthen the protein-protein interactions [21].

### Surface plasmon resonance analysis

Surface plasmon resonance analysis was performed with Biacore X100 system (GE Healthcare). Recombinant CRM1 and p53 protein were immobilized on an activated CM5 dextran chip (GE Healthcare, BR100399), respectively. The binding of TPR at different concentrations in the presence or absence of TLNC1 was performed with PBS containing 500 mM NaCl (pH 7.4) and 0.1% Tween 20. The flow rate was set at 30 μL/min. The binding kinetics was analyzed by BIAevaluation software using the 1:1 L binding model.

### Statistical analysis

Statistical analyses were conducted and graphics were generated using Prism version 9 (GraphPad Software). The association of TLNC1 and p21 mRNA with overall and disease-specific mortality of liver cancer patients was evaluated by Cox proportional hazards model. Univariate analyses were performed with Pearson χ^2^ and Fisher’s exact test. Patient survival between subgroups was compared by log-rank test. Survival rates were calculated by Kaplan-Meier method. All statistical tests were two-sided, and *p* < 0.05 was considered statistically significant. All quantitative data were recorded as mean ± standard error of measurement (SEM).

## Results

### TLNC1 is highly expressed in liver cancer tissues and correlated with patient prognosis

**Fig. 1:**
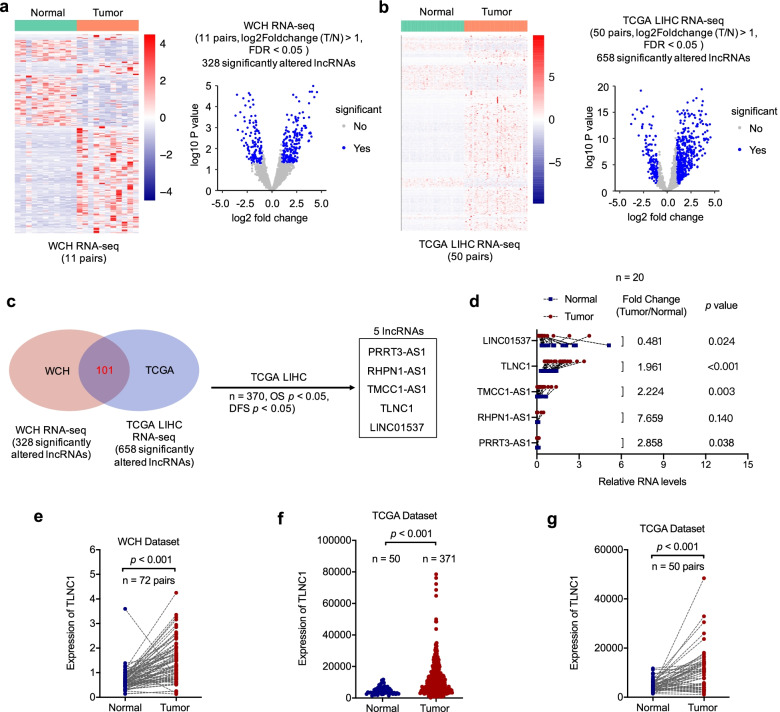
TLNC1 is upregulated in liver cancer tissues. **a** Clustering and volcano plot of differential lncRNA profiles between liver cancer tissues and adjacent normal tissues of west china hospital (WCH) cohort (*n* = 11). **b** Clustering and volcano plot of differential lncRNA profiles between liver cancer tissues and adjacent normal tissues of TCGA LIHC cohort (*n* = 50). **c** Venn plot shows the overlapping between differentially expressed lncRNAs of WCH and TCGA cohorts. The box indicates the differentially expressed lncRNAs that are correlated with OS and DFS of liver cancer patients according to the TCGA LIHC dataset. **d** Results from qRT-PCR validating the expression levels of 5 lncRNAs in 20 paired liver cancer and normal tissues. **e** Quantification of TLNC1 expression in 72 paired liver cancer and normal tissues of WCH cohort. **f** Quantification of TLNC1 expression in liver cancer tissues (*n* = 371) and normal tissues (*n* = 50) of TCGA LIHC cohort. **g** Quantification of TLNC1 expression in 50 paired liver cancer and normal tissues of TCGA LIHC cohort

To find lncRNAs that are associated with the development and progression of liver cancer, we performed RNA-seq with 11 paired liver cancer and adjacent normal liver tissues and identified 328 significant altered lncRNAs (Fig. 1a). To narrow the pool of candidate lncRNAs, we first utilized the RNA-seq data of TCGA LIHC cohort (50 pairs) and identified 658 significant altered lncRNAs (Fig. 1b). With the intersection of WCH and TCGA cohorts, 101 significant altered lncRNAs were identified, which were subsequently subjected to survival analysis based on the overall survival (OS) and disease-free survival (DFS) data of TCGA LIHC cohort. After survival analysis, we successfully identified 5 candidate lncRNAs, which were significantly differentially expressed between tumor and normal tissues, as well as significantly correlated with both OS and DFS of liver cancer patients (Fig. 1c). Next, the expression levels of these candidate lncRNAs were validated with 20 pairs of tissue samples by qRT-PCR. Based on the expression level data, TLNC1 was selected as the candidate lncRNA for further study because it was the most abundant lncRNA among the aforementioned 5 candidate lncRNAs (Fig. 1d). Moreover, we enlarged the sample size (*n* = 72) to confirm that TLNC1 was significantly highly expressed in liver cancer tissues (Fig. 1e). Consistently, similar results were also observed in TCGA LIHC cohort (Fig. 1f and g).

As listed in the National Center for Biotechnology Information database, the TLNC1 gene has only one annotated transcript (https://www.ncbi.nlm.nih.gov/; Fig. S1a). After analysis with the open reading frame finder (https://www.ncbi.nlm.nih.gov/orffinder/; Fig. S1b) and Coding-Potential Assessment Tool (CPAT) (Fig. S1c) [22], both software concluded that TLNC1 was with limited protein-coding potential. Next, fluorescence in situ hybridization (FISH) and RT-qPCR against TLNC1 showed the predominant nuclear distribution of TLNC1 (Fig. S2a and b).

Collectively, these findings demonstrated that TLNC1 is an abundant lncRNA significantly upregulated in hepatoma and correlated with patient prognosis.

### Overexpression of TLNC1 promotes growth and metastasis of liver cancer cells both in vitro and in vivo

**Fig. 2:**
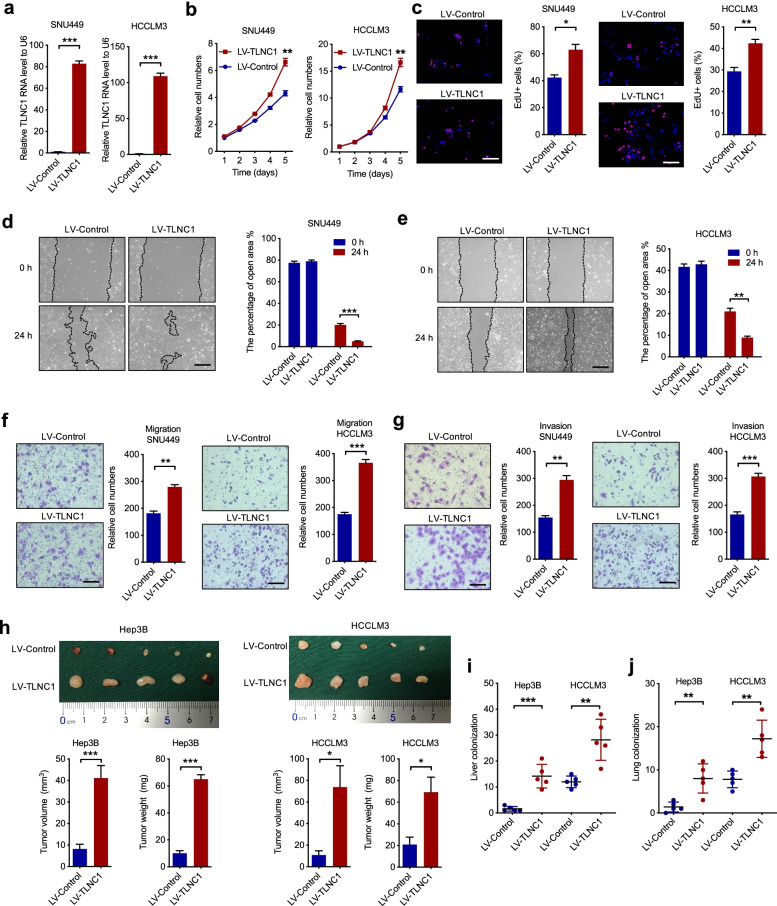
TLNC1 promotes tumor growth and metastasis of hepatoma cells. **a** qPCR quantification of the expression levels of TLNC1 in SNU449 and HCCLM3 cells stably transfected with lentivirus containing control or TLNC1 vectors. **b** Cell viability of indicated SNU449 and HCCLM3 cells was measured by CCK-8 assay. **c** Cell proliferation of indicated SNU449 and HCCLM3 cells was examined by EdU assay. **d-e** Cell migration of indicated SNU449 and HCCLM3 cells was measured by wound healing assay. **f-g** Cell migration and invasion of indicated SNU449 and HCCLM3 cells was measured by transwell migration and matrigel invasion assays. The data are the means ± SEM and are representative of three independent experiments. **h** Effects of TLNC1 overexpression in indicated Hep3B and HCCLM3 cells on subcutaneous tumor growth. Tumor volumes and tumor weights were measured after the mice were killed (*n* = 5). **i** The number of tumor nodules formed in the livers of orthotopic-implantation models inoculated with indicated hepatoma cells was indicated in the bar graph (*n* = 5). **j** The number of metastatic foci formed in the lungs of lung metastasis models inoculated with indicated hepatoma cells was indicated in the bar graph (*n* = 5). *p < 0.05, **p < 0.01, ***p < 0.001

To ascertain that whether TLNC1 functions as a tumorigenic lncRNA, we constructed two TLNC1-overexpression hepatoma cell lines, SNU449 and HCCLM3 cells (Fig. 2a). CCK-8 and EdU assays showed that overexpression of TLNC1 significantly elevated cell proliferation of SNU449 and HCCLM3 cells (Fig. 2b and c). In addition, we demonstrated that the cell motility was increased in TLNC1-overexpression hepatoma cells in comparison to indicated controls (Fig. 2d-g). To validate the oncogenic function of TLNC1 in vivo, we first constructed subcutaneous xenograft hepatoma mouse models and found that enforced expression of TLNC1 drastically elevated the volumes and weights of xenograft tumors (Fig. 2h). In addition, liver orthotopic-implantation models and lung metastasis models were established to evaluate the effects of TLNC1 overexpression on tumor metastasis. The in vivo imaging system (IVIS) imaging results suggested that TLNC1-overexpression group showed stronger bioluminescence signal intensities in liver (Fig. S3a). In the meantime, overexpression of TLNC1 significantly promoted liver colonization of hepatoma cells (Fig. S3b and Fig. 2i). Consistently, TLNC1-overexpression group showed more lung metastatic foci in lung metastasis models (Fig. S3c and Fig. 2j). To sum up, these data indicated that TLNC1 overexpression promotes the cell proliferation and metastasis of liver cancer.

### TLNC1 is essential for tumor progression and metastasis of liver cancer

**Fig. 3:**
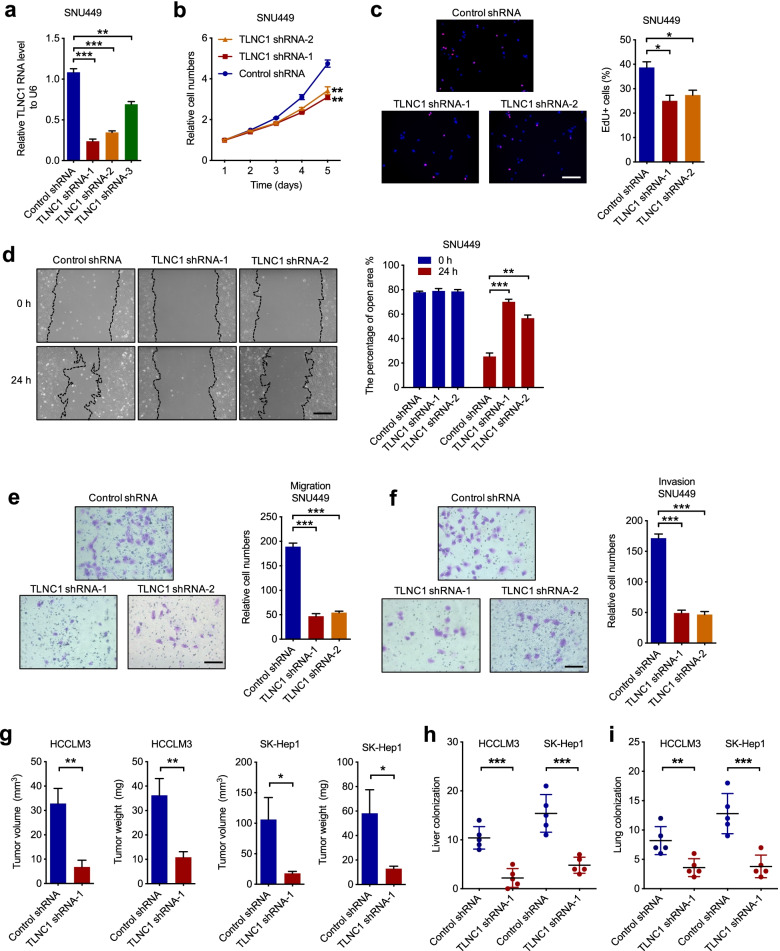
Knockdown of TLNC1 represses tumor growth and metastasis of hepatoma cells. **a** qPCR quantification of the expression levels of TLNC1 in SNU449 cells transfected with control shRNA or shRNAs targeting TLNC1. **b** Cell viability of indicated SNU449 cells was measured by CCK-8 assay. **c** Cell proliferation of indicated SNU449 cells was examined by EdU assay. **d** Cell migration of indicated SNU449 cells was measured by wound healing assay. **e-f** Cell migration and invasion of indicated SNU449 cells were measured by transwell migration and matrigel invasion assays, respectively. The data are the means ± SEM and are representative of three independent experiments. **g** Effects of TLNC1 knockdown in HCCLM3 and SK-Hep1 cells on subcutaneous tumor growth. Tumor volumes and tumor weights were measured after the mice were killed (*n* = 5). **h** The number of tumor nodules formed in the livers of orthotopic-implantation models inoculated with indicated hepatoma cells was indicated in the bar graph (*n* = 5). **i** The number of metastatic foci formed in the lungs of lung metastasis models inoculated with indicated hepatoma cells was indicated in the bar graph (*n* = 5). *p < 0.05, **p < 0.01, ***p < 0.001

To confirm the function of TLNC1 in tumor progression and metastasis of liver cancer, we silenced TLNC1 using 3 independent short hairpin RNAs (shRNAs) in SNU449 and HCCLM3 cells (Fig. 3a and Fig. S4a) and chose 2 shRNAs with better knockdown efficacy for subsequent experiments. It was shown that silencing of TLNC1 inhibited cell growth of SNU449 and HCCLM3 cells (Fig. 3b, c and Fig. S4b, c). In the meantime, the cell motility was dramatically downregulated in TLNC1-knockdown hepatoma cell lines compared with indicated controls (Fig. 3d-f and Fig. S4d-f). To determine whether TLNC1 is required for the growth and metastasis of hepatoma in vivo, we first constructed subcutaneous xenograft hepatoma mouse models and showed that silencing of TLNC1 decreased the volumes and weights of xenograft tumors (Fig. S4g and Fig. 3g). In addition, liver orthotopic-implantation models and lung metastasis models were utilized to validate the function of TLNC1 silencing on tumor metastasis. The IVIS imaging results suggested that TLNC1-knockdown group showed weaker bioluminescence signal intensities in liver (Fig. S4h). Meanwhile, knockdown of TLNC1 markedly inhibited liver colonization of hepatoma cells (Fig. S4i and Fig. 3h). Consistently, TLNC1-knockdown group showed less lung metastatic foci in lung metastasis models (Fig. S4j and Fig. 3i). Taken together, our results suggested that TLNC1 is needed for the growth and metastasis of liver cancer cells.

### TLNC1 regulates multiple signaling pathways associated with cell growth and metastasis

**Fig. 4:**
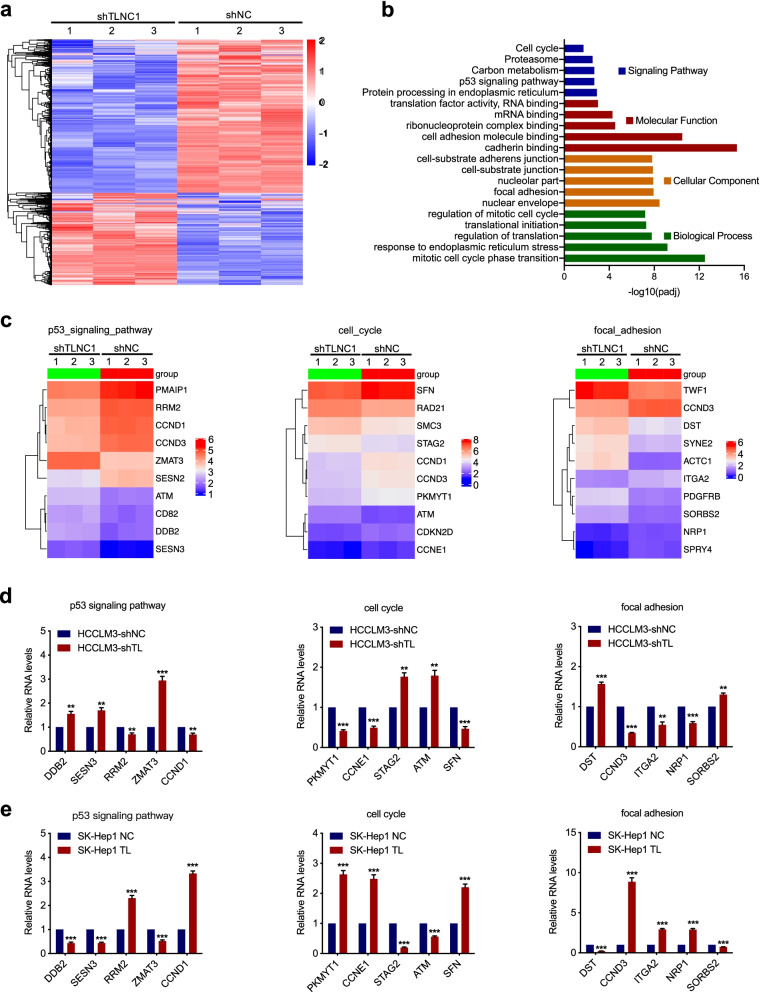
TLNC1 induces deregulation of the genes involved in cell proliferation and metastasis in hepatoma cells. **a** Heatmap of differentially expressed genes from HCCLM3 cells transfected with control shRNA or shRNA targeting TLNC1. Red indicates upregulated genes and blue indicates downregulated genes. **b** Functional annotation by KEGG and GO analysis revealed genes involved in p53 signaling pathway, cell cycle, and cell adhesion process. **c** Heatmap of differentially expressed genes upon TLNC1 knockdown enriched in p53 signaling pathway, cell cycle and focal adhesion process. **d-e** qRT-PCR results confirmed the differentially expressed genes selected from functional annotation analysis in indicated HCCLM3 (TLNC1 knockdown) and SK-Hep1 (TLNC1 overexpression) cells. The data are the means ± SEM and are representative of three independent experiments. **p < 0.01, ***p < 0.001

To explore the underlying mechanism of TLNC1-mediated tumor progression and metastasis of liver cancer, we performed RNA-seq to identify the genes regulated by knockdown of TLNC1 and identified 4250 up-regulated and 2997 down-regulated genes (adjusted *p* < 0.05) in TLNC1 knockdown HCCLM3 cells compared with controls (Fig. 4a). Kyoto Encyclopedia of Genes and Genomes (KEGG) pathway analysis showed that p53 signaling pathway and cell cycle were among the most remarkable pathways enriched. Gene Ontology (GO) analysis implied that TLNC1 could regulate a series of biological processes that are associated with tumor progression, such as cell adhesion, focal adhesion and regulation of mitotic cell cycle (Fig. 4b). After that, top 10 genes with the most prominent fold change were identified in p53 signaling pathway, cell cycle and focal adhesion gene sets, respectively (Fig. 4c). To verify the data of RNA-seq, we examined the mRNA levels of 5 genes from each gene sets with qRT-PCR. As shown in Fig. 4d and e, modulation of TLNC1 indeed regulates the expression levels of the genes related to tumor growth and metastasis.

### TLNC1 interacts with TPR to regulate p53 signaling pathway

To further understand the detailed mechanism of TLNC1-regulated tumor growth and metastasis, we then used biotin-labeled RNA pull-down and mass spectrometry (MS) to identify proteins that interacted with TLNC1 in hepatoma cells. As we have proved that TLNC1 mainly localized in the nucleus, we only extracted nuclear proteins for RNA pull-down assay. A total of 393 proteins were identified as TLNC1-interacting proteins. Of them, translocated promoter region (TPR), a component of the nuclear pore complex (NPC), had the highest score (Fig. S5a). Interestingly, TPR has been identified as a member of the nuclear export complex of p53 [14]. Since p53 is a well-established regulator of tumor growth and metastasis [23], therefore, based on the intersection of RNA-seq and MS data, we hypothesized that TLNC1 might regulate p53 signaling pathway through interacting with TPR. To verify this hypothesis, we first validated the interaction between TLNC1 and TPR. As shown in Fig. S5b and c, TLNC1 could indeed interact with TPR.

**Fig. 5:**
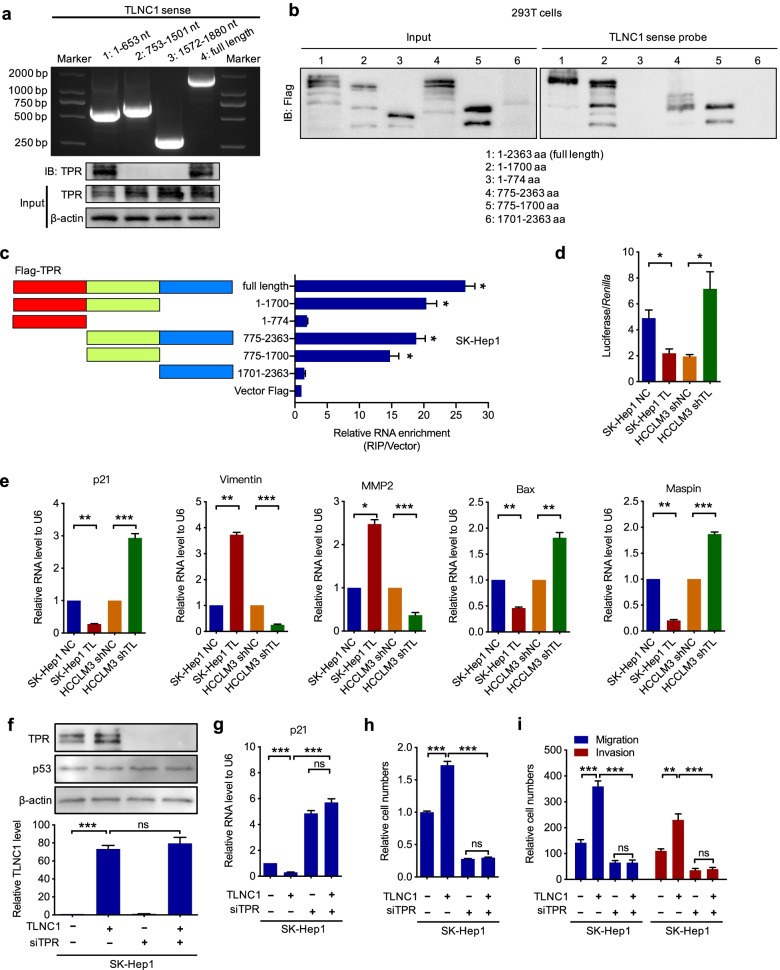
TLNC1 interacts with TPR and represses the transcriptional activity of p53. **a** Validation of nucleotide sequences in TLNC1 that bind to TPR. Upper panel, nucleotide electrophoresis showing the sizes of full-length and truncated TLNC1. Lower panel, immunoblot analysis of TPR in protein samples pulled down by full-length or truncated TLNC1. **b** Immunoblotting of the full-length or truncated Flag-TPR in the lysates from 293 T cells transfected with the indicated vectors, or in the retrieved proteins by the biotinylated TLNC1 probe from the lysates of 293 T cells transfected with the indicated vectors. **c** Validation of amino acid sequences in TPR that bind to TLNC1. Left panel, diagrams of full-length and truncated Flag-TPR. Right panel, qRT-PCR analysis after RIP assays of SK-Hep1 cells transfected with full-length and truncated Flag-TPR. **d** Dual luciferase assays for SK-Hep1 cells transfected with control or TLNC1 vectors and HCCLM3 cells transfected with control shRNA or shRNA targeting TLNC1. **e** qRT-PCR analysis for the expression of downstream targets of p53 signaling in indicated SK-Hep1 (TLNC1 overexpression) and HCCLM3 (TLNC1 knockdown) cells. **f** Above, immunoblot analysis of TPR and p53 in indicated SK-Hep1 cells treated with or without siTPR. Below, qRT-PCR detection of TLNC1 in indicated SK-Hep1 cells treated with or without siTPR. **g** qRT-PCR analysis for the expression of p21 in indicated SK-Hep1 cells. **h** Cell viability of indicated SK-Hep1 cells was measured by CCK-8 assay at 5 days post seeding. **i** Cell migration and invasion of indicated SK-Hep1 cells were measured by transwell migration and matrigel invasion assays, respectively. The data are the means ± SEM and are representative of three independent experiments. *p < 0.05, **p < 0.01, ***p < 0.001

Next, we sought to identify the interacting sites between TLNC1 and TPR. According to the RNA secondary structure prediction software, RNAfold [24], we proposed 3 potential parts that might interact with TPR (Fig. S5d). Based on the prediction, we constructed 3 truncated TLNC1 fragments and showed that 1–653 nucleotides were required for the interaction between TLNC1 and TPR (Fig. 5a). After that, we performed RNA pull-down and RIP assays to show that the 775–1700 amino acids of TPR were responsible for the interaction of TPR with TLNC1 (Fig. 5b and c).

To determine whether TLNC1 is able to regulate p53 signaling pathway, we performed dual luciferase reporter assay with a vector containing p21 promoter, a well-known transcriptional target of p53 [20]. It was shown that TLNC1 overexpression dramatically downregulated the transcriptional activity of p53 in hepatoma cells (Fig. 5d). Consistently, the mRNA levels of p53 target genes, such as p21, Vimentin, MMP2, Bax, and Maspin, could be modulated by TLNC1 (Fig. 5e). To further ascertain that TLNC1 regulated p53 signaling pathway via TPR, we knockdown TPR with siRNA in TLNC1 overexpression hepatoma cells (Fig. 5f). As shown in Fig. 5g-i, knockdown of TPR blocked TLNC1 overexpression-mediated downregulation of p21, as well as the tumorigenic functions of TLNC1. In the meantime, knockdown of TPR also markedly blocked the effect of TLNC1 overexpression on the expression levels of focal adhesion-related genes (Fig. S6).

Taken together, these results demonstrated that TLNC1 could regulate p53 signaling pathway through interaction with TPR.

### TLNC1 promotes the cytoplasmic translocation of p53 through interaction with TPR

**Fig. 6:**
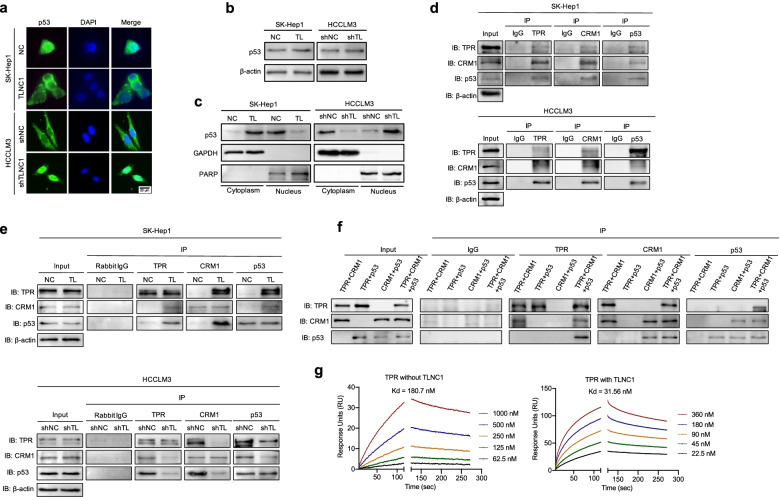
TLNC1 represses nuclear translocation of p53 through interaction with TPR. **a** p53 expression in indicated SK-Hep1 and HCCLM3 cells, as detected by an immunofluorescence assay. The merged images show overlays of p53 (green) and nuclear staining by DAPI (blue); scale bar: 20 μm. **b** Immunoblot analysis of p53 in indicated SK-Hep1 and HCCLM3 cells. **c** p53 expression in cytoplasmic and nuclear fractions, as detected by immunoblot analysis. GAPDH was used as a loading control for the cytoplasmic fraction, and PARP was used as a loading control for the nuclear fraction. **d** Co-immunoprecipitation among TPR, CRM1 and p53 in SK-Hep1 and HCCLM3 cells, as detected by immunoblot analysis. **e** Co-immunoprecipitation among TPR, CRM1 and p53 in indicated SK-Hep1 (TLNC1 overexpression) and HCCLM3 (TLNC1 knockdown) cells, as detected by immunoblot analysis. **f** Co-immunoprecipitation among recombinant TPR, CRM1 and p53 proteins, as detected by immunoblot analysis. The data are the representative of three independent experiments. **g** Surface plasmon resonance analysis of the binding of CRM1 with increasing concentrations of recombinant TPR in the presence or absence of TLNC1

In order to exert the function of transcription factor, p53 has to translocate from cytoplasm to nucleus [12]. To understand the mechanism how TLNC1 regulates p53 signaling pathway, we examined the subcellular localization of p53 upon TLNC1 overexpression or knockdown. As shown in Fig. 6a, TLNC1 overexpression drastically stimulated the cytoplasmic translocation of p53. Immunoblots further confirmed this observation that although the total protein levels of p53 remained unchanged upon TLNC1 overexpression, the amount of p53 in the nucleus decreased significantly (Fig. 6b and c).

According to previous studies, TPR can form a complex with chromosome maintenance region 1 (CRM1) to mediate the nuclear export of p53 [14]. To determine whether TLNC1 facilitated the nuclear export of p53 through modulation of the TPR-CRM1-p53 complex, we examined the interactions among TPR, CRM1 and p53 with co-IP. As shown in Fig. 6d, TPR could interact with CRM1 and p53 in wild-type hepatoma cells. Interestingly, overexpression of TLNC1 enhanced the interaction of the TPR-CRM1-p53 complex, while knockdown of TLNC1 repressed the interaction of the TPR-CRM1-p53 complex (Fig. 6e). To further discover how TLNC1 promotes the complex formation, we first examined the protein-protein interaction with purified recombinant proteins to exclude the interference of other proteins in cell lysates. As shown in Fig. 6f, TPR was shown to directly bind to CRM1, and CRM1 could directly bind to p53; however, TPR could not directly bind to p53. Next, surface plasmon resonance assay revealed that in the presence of TLNC1, the affinity between TPR and CRM1 increased significantly (Fig. 6g). Meanwhile, no direct interaction between TPR and p53 was confirmed (Fig. S7).

Collectively, these data demonstrated that TLNC1 induces the nuclear export of p53 through interaction with TPR and upregulates the affinity between TPR and CRM1.

### Upregulation of TLNC1 predicts poor survival in liver cancer patients

**Fig. 7:**
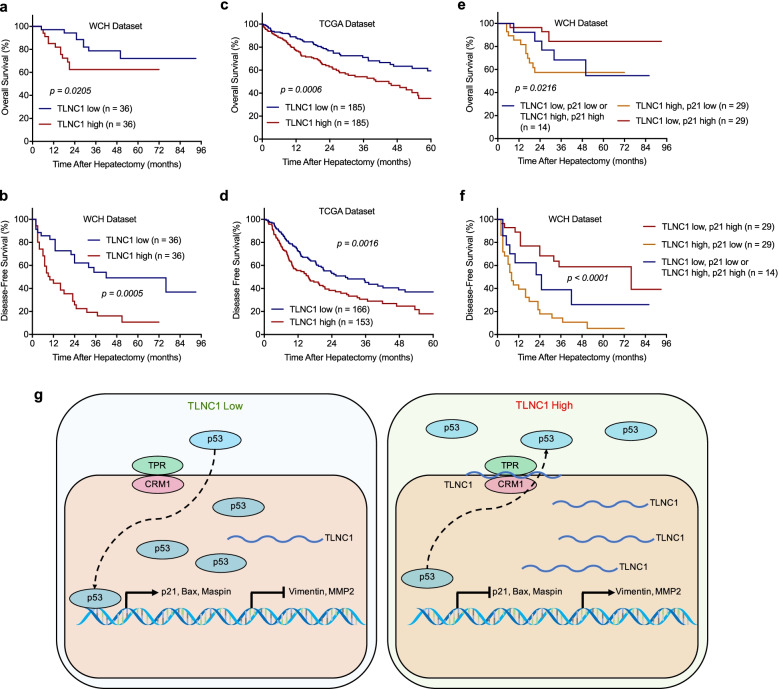
High levels of TLNC1 combined with low levels of p21 is associated with poor prognosis of liver cancer patients. **a-b** Kaplan-Meier curves for overall survival and disease-free survival of patients from the West China Hospital (WCH) dataset with high or low TLNC1 expression levels. **c-d** Kaplan-Meier curves for overall survival and disease-free survival of patients from the TCGA dataset with high or low TLNC1 expression levels. **e-f** Kaplan-Meier curves for overall survival and disease-free survival based on the combination of TLNC1 and p21 mRNA expression levels. Liver cancer patients were divided into three groups based on TLNC1 and p21 mRNA expression: group 1 (*n* = 29): high TLNC1 and low p21 expression; group 2 (*n* = 29): low TLNC1 and high p21 expression; group 3 (*n* = 14): low TLNC1 and p21 expression or high TLNC1 and p21 expression. **g** A schematic model showing the function of TLNC1 in regulating p53 signaling pathway. Elevated TLNC1 levels promote the nuclear export of p53 through strengthening the protein-protein interactions among p53, CRM1 and TPR. TLNC1 represses the transcription of a series of tumor suppressors, including p21, Bax and Maspin, meanwhile activates the transcription of genes associated with metastasis, such as Vimentin and MMP2, which ultimately contributes to the promotion of tumor growth and metastasis of hepatoma

To further investigate the clinical significance of TLNC1 expression in liver cancer, 72 liver cancer patients were divided into 2 groups according to the expression levels of TLNC1: the high TLNC1 expression group (*n* = 36) and the low TLNC1 expression group (*n* = 36). The basic demographic data for these patients were listed in Table S2. As revealed by correlation analysis, TLNC1 expression was significantly related to cirrhosis (*p* = 0.026) (Table S3). Univariable analysis revealed that both the OS of high TLNC1 expression group (hazard ratio (HR), 2.28; 95% confidence interval (CI), 0.92 to 5.63) and the DFS of high TLNC1 expression group (HR, 1.06; 95% CI, 0.59 to 1.93) were worse than the low TLNC1 expression group (*p* < 0.05) (Table S4). Likewise, survival curves of both the West China Hospital (WCH) and TCGA datasets revealed that TLNC1 expression was negatively correlated with patient prognosis (Fig. 7a-d).

To verify that TLNC1 could regulate p53 signaling pathway in human hepatoma, we detected the expression levels of p21 mRNA and found that the mRNA levels of p21 were negatively correlated with the expression levels of TLNC1 (*r* = − 0.6249, *p* < 0.0001) (Fig. S8). These data proved that TLNC1 could induce the nuclear export of p53 and repress the transcription of p21. To evaluate the clinical potential of TLNC1-p53 axis, we then analyzed the prognostic potential of TLNC1 and p21 in combination. Strikingly, it was shown that patients with low expression of TLNC1 and high p21 had the best prognosis; on the contrary, patients with high expression of TLNC1 and low p21 had the worst prognosis (Fig. 7e and f). In summary, these data demonstrated that the combination of TLNC1 and p21 could be utilized as a prognostic factor of liver cancer patients.

## Discussion

A high rate of recurrence and metastasis, which directly contributes to poor prognosis, is among the main challenges in treating liver cancer [3]. In order to develop effective strategies to block the recurrence and metastasis of liver cancer, it is critical to investigate the detailed molecular mechanisms underlying the biological processes related to the recurrence and metastasis of hepatoma. Recently, a variety of lncRNAs have been reported to play vital roles in tumor progression and metastasis of many cancer types. For example, lncRNA PVT1 promotes tumor progression by regulating the miR-143/HK2 axis in gallbladder cancer [25]. LncRNA BCRT1 promotes breast cancer progression by targeting miR-1303/PTBP3 axis [5]. LncRNA NEAT1 activates Wnt/β-catenin signaling and promotes colorectal cancer progression via interacting with DDX5 [26]. However, the biological functions and molecular mechanisms within the recurrence and metastasis of liver cancer remain largely unknown. In this study, we integrated the tumor vs normal RNA-seq data of our own (WCH cohort) with TCGA LIHC cohort to identify the significantly altered lncRNAs in liver cancer. The background information of liver cancer patients in WCH cohort and TCGA cohort are quite different, such as race, etiology and life style, etc. Therefore, the intersection of the two cohorts might be able to identify key lncRNAs that are commonly shared by all the populations. More importantly, we took overall and disease-free survival time into account during the identification of candidate lncRNAs, which enabled us to find out the lncRNAs closely related to patient prognosis, especially recurrence and metastasis. Utilizing the aforementioned approach, we successfully identified a series of candidate lncRNAs and chose TLNC1, a significantly upregulated lncRNA with the highest abundance, for further study.

Here, we revealed the role of TLNC1, which was also known as linc01134, in tumor growth and metastasis of liver cancer. Previously, the transcription of linc01134 was shown to be enhanced by Yin Yang-1 (YY1). Reciprocally, linc00134 could act as miR-324-5p sponge and interact with insulin-like growth factor 2 mRNA binding protein 1 (IGF2BP1) to increase the stability of YY1 mRNA expression. This positive feedback loop has been proved to promote the progression of HCC [16]. In addition, linc00134 was also shown to promote HCC metastasis via activating AKT1S1 and NF-κB signaling [17]. Another study proved that linc01134 could accelerate HCC progression by sponging miRNA-4784 and downregulating structure specific recognition protein 1 (SSRP1) [18]. The prognostic value of linc00134 was documented along with the other 5 upregulated lncRNAs in HCC [27]. Last but not least, a recent published paper also demonstrated that linc01134 was able to confer oxaliplatin resistance through SP1-induced p62 transcription in HCC [19]. Collectively, these studies demonstrated that linc00134 was a key lncRNA in HCC, suggesting that this lncRNA is worthy of in-depth study. Although our study was not the first one to reveal the oncogenic functions of linc00134 in liver cancer, it was first study to combine the high-throughput data of an independent cohort of the Chinese population and TCGA cohort, instead of using TCGA LIHC cohort alone, to screen the potential key lncRNAs associated with the progression of liver cancer. The identification approach in our study was significantly more rational, which demonstrated the universality among different populations of linc00134 and thus widened the potential application range of this key lncRNA. More importantly, our study was the first one to comprehensively dissect the interacting proteins of linc01134. Previous studies mainly focused on the competing endogenous RNA (ceRNA) function of linc00134 on miRNAs, while our study successfully identified a series of linc01134-interacting proteins and revealed a novel signaling axis, the linc01134-TPR-CRM1-p53 axis. Last but not least, our study was the only study that utilized both knockdown and overexpression modulation to investigate the biological functions of linc01134 in tumor growth and metastasis both in vitro and in vivo, thus it was so far the most comprehensive study of linc01134. Interestingly, the multi-omics data of our study also confirmed a couple of findings of the previous observations. For example, according to our KEGG analysis, TLNC1 is able to modulate several pathways associated with oxidative stress regulation, such as oxidative phosphorylation and glutathione metabolism, which is consistent with the finding of the paper published by Ma et al. [19]. More importantly, based on our study and the previous studies, it is reasonable to conclude that TLNC1 is able to promote the progression and metastasis, as well as confer drug resistance in liver cancer. To exert its functions, TLNC1 serves as sponges of miRNAs [16, 18], or interacts with gene promoters [17] and proteins, such as SP-1 [19] and TPR, to regulate a series of signaling pathways including NF-κB and p53 signaling pathway. Although the roles and molecular mechanisms of TLNC1 have been partially revealed in liver cancer, more questions of TLNC1 remain unsolved, such as what roles does TLNC1 play in cancer stem cell or immunotherapy?

The main novelty of our study was that we thoroughly dissected and analyzed the alterations of downstream gene expressions of TLNC1 by performing RNA-seq. In the meantime, we also performed RNA pull-down coupled with MS analysis to identify the interacting proteins of TLNC1. Based on the RNA-seq and MS data, we proposed and demonstrated a novel mechanism of TLNC1-regulated nuclear export process of p53. Previously, p53 has been shown to be exported by the nuclear pore complex consisting of TPR and CRM1 [14, 15]; however whether p53 directly interacts with TPR or CRM1 was unknown. In this study, we utilized recombinant proteins to show that p53 could directly interact with CRM1, but not TPR, suggesting that CRM1 is a vital mediator for the interaction between TPR and p53. More importantly, we demonstrated that TLNC1 could reinforce the interaction between TPR and CRM1 via binding to TPR, indicating that TLNC1 is a key regulator of the interaction between TPR and CRM1, which subsequently functions as a critical regulator of p53 signaling pathway. Since p53 is the most significantly mutated gene in liver cancer [28], indicating the vital role of p53 in the development and progression of liver cancer, our study provide potential therapeutic targets for liver cancer by revealing a novel signaling axis of p53 that is governed by TLNC1. Moreover, the high-throughput data of our study will inspire more in-depth investigations of the biological functions and mechanisms of TLNC1.

To sum up, our study showed that TLNC1 was able to promote tumor growth and metastasis of hepatoma both in vitro and in vivo. Mechanistically, we demonstrated that TLNC1 could interact with TPR to strengthen the interaction between TPR and CRM1, which then promote the nuclear export of p53, leading to the downregulation of a series of tumor suppressors and the upregulation of many oncogenes, finally contribute to the induction of tumor growth and metastasis of liver cancer. Moreover, we proved that TLNC1 was closely correlated with both the OS and DFS of hepatoma patients and the combination of TLNC1 and p21, a p53 target gene, could serve as a prognostic biomarker for hepatoma patients.

## Supplementary Information

**Additional file 1.**

## Data Availability

All data generated or analyzed during this study are included either in this article or in the supplementary information files.
