# Supplementary material for: Long noncoding RNA TLNC1 promotes the growth and metastasis of liver cancer via inhibition of p53 signaling
Source: Mol Cancer. 2022 Apr 27;21:105. doi: 10.1186/s12943-022-01578-w (PMC9044722; doi:10.1186/s12943-022-01578-w)
Supplement: Supplementary file 1 — Additional file 1. [file 12943_2022_1578_MOESM1_ESM.docx]

**Supplementary Figure Legends**

**Fig. S1** Characterization of human TLNC1 as long non-coding RNA. **a** Schematic diagram of the genomic locus of TLNC1 (NCBI gene, https://www.ncbi.nlm.nih.gov/). **b** Open reading frame (ORF) Finder software prediction (the ORF finder, https://www.ncbi.nlm.nih.gov/orffinder/) for the protein-coding potential of TLNC1. **c** Coding-Potential Assessment Tool prediction for the protein-coding potential of TLNC1.


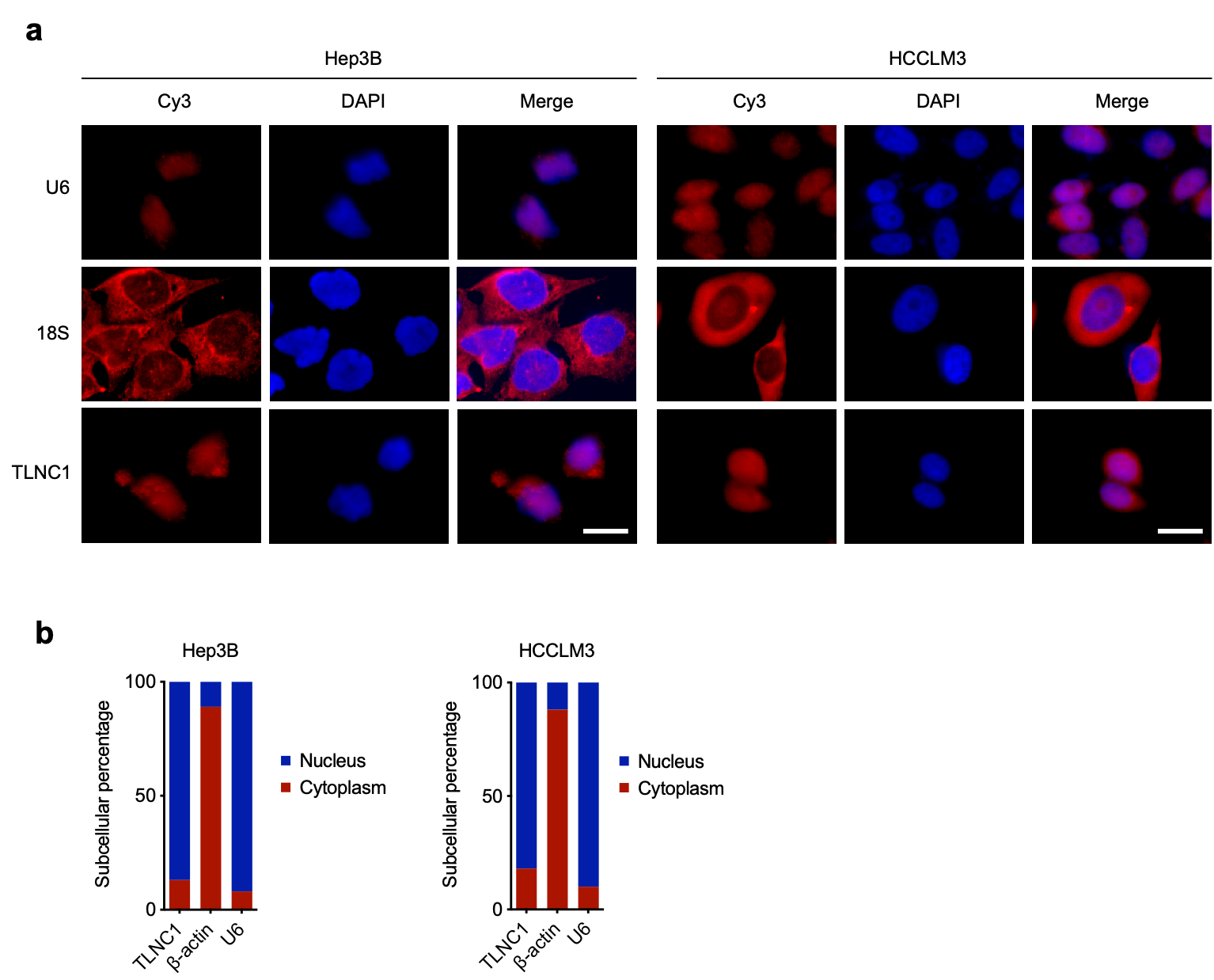


**Fig. S2** Subcellular location of TLNC1. **a** RNA FISH for TLNC1 in indicated liver cancer cells. Nuclei were stained with DAPI. 18S and U6 were applied as positive controls in the cytoplasm and nucleus, respectively. Scale bar, 10 μm. **b** Cytoplasmic and nuclear RNA fractions were isolated by using the PARISTM Kit (Invitrogen, CA, USA) according to manufacturer’s protocol, followed by qRT-PCR analysis of the expression levels of TLNC1. β-actin and U6 were applied as positive controls in the cytoplasm and nucleus, respectively. The data are the means ± SEM and are representative of three independent experiments.


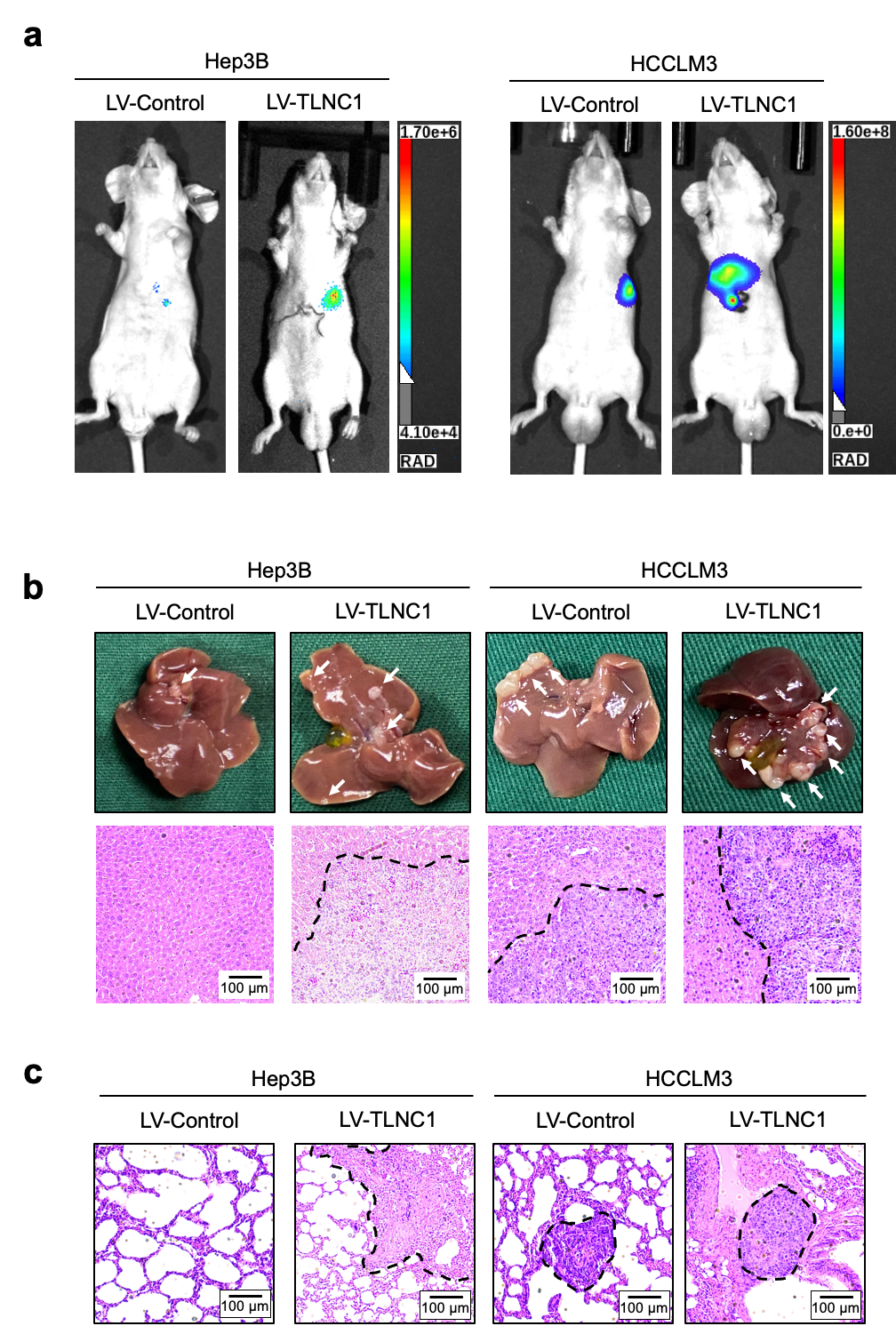


**Fig. S3** Effects of TLNC1 overexpression in indicated hepatoma cells on liver or lung metastasis. **a** Representative bioluminescent images of liver tumors from orthotopic-implantation models inoculated with indicated Hep3B and HCCLM3 cells. **b** Representative gross and HE images of liver tumors from orthotopic-implantation models. **c** Representative HE images of lung metastatic foci formed in the lungs of lung metastasis models inoculated with indicated Hep3B and HCCLM3 cells.


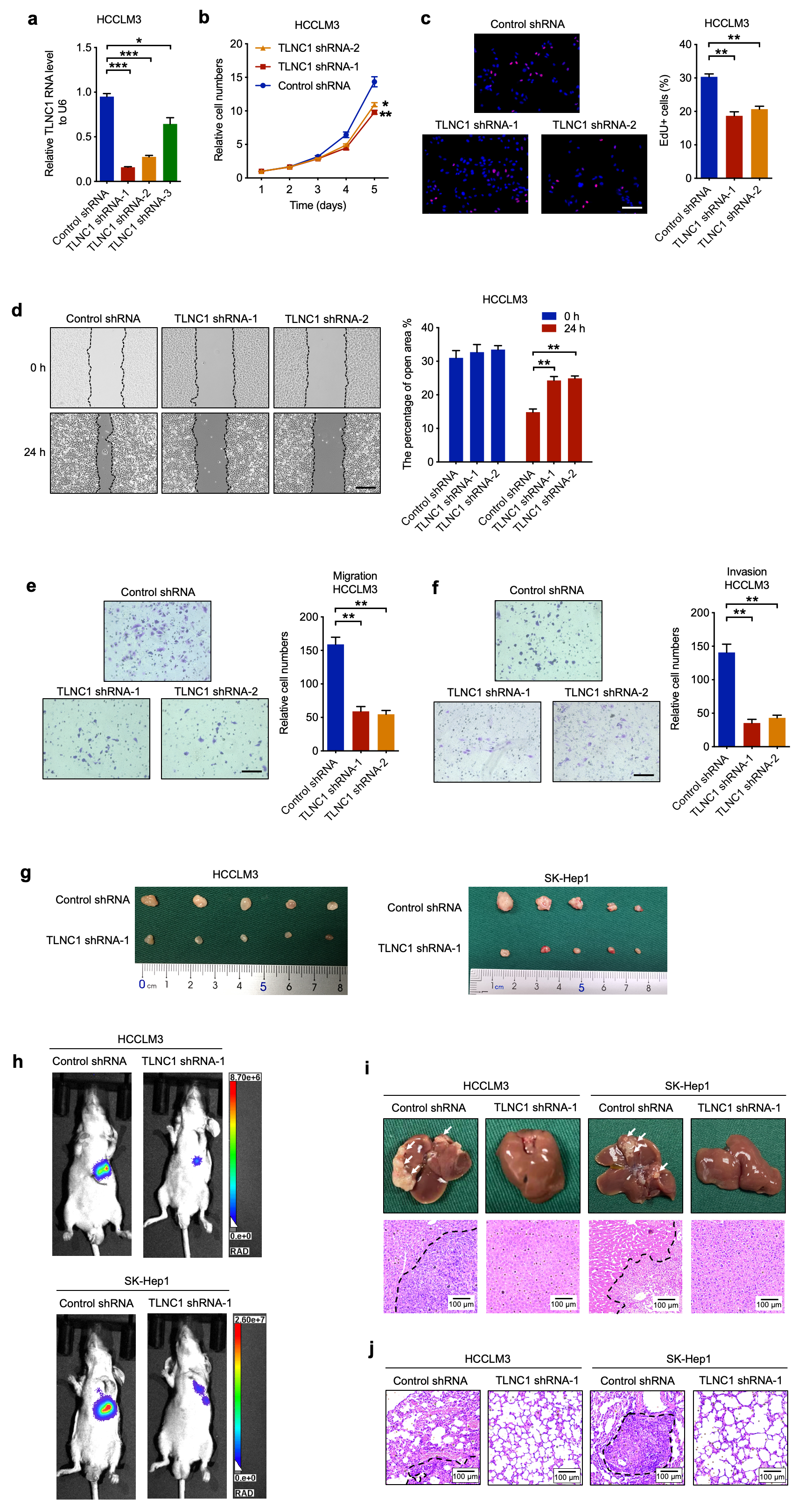


**Fig. S4** Knockdown of TLNC1 represses tumor growth and metastasis of hepatoma cells. **a** qPCR quantification of the expression levels of TLNC1 in HCCLM3 cells transfected with control shRNA or shRNAs targeting TLNC1. **b** Cell viability of indicated HCCLM3 cells was measured by CCK-8 assay. **c** Cell proliferation of indicated HCCLM3 cells was examined by EdU assay. **d** Cell migration of indicated HCCLM3 cells was measured by wound healing assay. **e-f** Cell migration and invasion of indicated HCCLM3 cells were measured by transwell migration and matrigel invasion assays, respectively. The data are the means ± SEM and are representative of three independent experiments. **g** Gross images showing the effects of TLNC1 knockdown in HCCLM3 and SK-Hep1 cells on subcutaneous tumor growth (n = 5). **h** Representative bioluminescent images of liver tumors from orthotopic-implantation models inoculated with indicated HCCLM3 and SK-Hep1 cells. **i** Representative gross and HE images of liver tumors from orthotopic-implantation models. **j** Representative HE images of lung metastatic foci formed in the lungs of lung metastasis models inoculated with indicated HCCLM3 and SK-Hep1 cells. *p < 0.05, **p < 0.01, ***p < 0.001.

**Fig. S5** Identification of TLNC1-interacting proteins. **a** Venn plot shows the number of proteins that can only be pulled down by TLNC1 sense biotin-labeled probe, but not the antisense probe. The table lists the top 5 potential TLNC1-interacting proteins with the highest score. **b-c** The TLNC1-TPR interaction was validated by RNA pull-down (b) and RNA immunoprecipitation (c) assays in SNU449 and HCCLM3 cells (b) and HCCLM3 cells (c), respectively. The data are the means ± SEM and are representative of three independent experiments. **d** Graphic illustration of predicted TLNC1 secondary structure (LNCipedia, http://www.lncipedia.org), and the truncation diagram of TLNC1 according to the stem-loop structure. *p < 0.05.

**Fig. S6** qRT-PCR results confirmed that knockdown of TPR markedly blocked the effect of TLNC1 overexpression on the expression levels of focal adhesion-related genes (DST, CCND3, ITGA2, NRP1 and SORBS2) in SK-Hep1 cells. The data are the means ± SEM and are representative of three independent experiments. ***p < 0.001.

**Fig. S7** Validation of the direct interaction between p53 and TPR. Surface plasmon resonance analysis of the binding of p53 with increasing concentrations of recombinant TPR in the absence of TLNC1.

**Fig. S8** Correlation analysis between TLNC1 and p21 mRNA expression in liver tumor tissues from patients with liver cancer (n = 72).

Supplementary table 1. The primer sequences used for real-time quantitative PCR.

| Primer Names | Sequences (5’-3’) |
| --- | --- |
| LncRNA TLNC1 Forward | GGACATGAACAGCAAATGCACA |
| LncRNA TLNC1 Reverse | TTCCTGGATGTCACCATGAAGG |
| NRP1 Forward | GGGAAAAGGGAGAGGAAGCC |
| NRP1 Reverse | ACTCCGCCTAGAGCTGTACA |
| SORBS2 Forward | CATGTTCCACCTCCAGTCCC |
| SORBS2 Reverse | AGGAGGCTGGTCTTTCATGC |
| ITGA2 Forward | ATTTCTTGAAGGCCCCGAGG |
| ITGA2 Reverse | CGGATAGTGCCCTGATGACC |
| SFN Forward | CTGCTGCCTCTGATCGTAGG |
| SFN Reverse | AAACATGGTCACACCCAGCA |
| STAG2 Forward | TGAGCTGCTGAAAGACTGGG |
| STAG2 Reverse | TGACATTCAGCCGCTTGTCT |
| CCNE1 Forward | TGGCGTTTAAGTCCCCTGAC |
| CCNE1 Reverse | AAGGCCGAAGCAGCAAGTAT |
| CCND1 Forward | AGCTGTGCATCTACACCGAC |
| CCND1 Reverse | TGTTTGTTCTCCTCCGCCTC |
| DDB2 Forward | CTGGCCTCTGCAATGGGTTA |
| DDB2 Reverse | CCTCTGCTTGCAGGACTTGA |
| DST Forward | GGCGTTGGAAAGAAGGAGGA |
| DST Reverse | GCCGCGTTATTTTCCCATCC |
| ATM Forward | TGCGTGCACTGAAAGAGGAT |
| ATM Reverse | AGCCAGAGGGAACAAAGTCG |
| CCND3 Forward | CAGCGCCTTTCCCAACTCTA |
| CCND3 Reverse | AAAACATGAGAGCCCCCAGG |
| SESN3 Forward | TGACAAGAGGACCAAGTGCC |
| SESN3 Reverse | CCGCAAGAAAGACTCCAGGT |
| RRM2 Forward | AAGAAGAAGGCAGACTGGGC |
| RRM2 Reverse | CCAGGCATCAGTCCTCGTTT |
| ZMAT3 Forward | GCCACCAGGTCTACAGGAAC |
| ZMAT3 Reverse | TGCTCCCCTCCCTTCGATAA |
| PKMYT1 Forward | AGCGGATGTGTTCAGTCTGG |
| PKMYT1 Reverse | CTGGCTCCAGCATCATGACA |
| Maspin Forward | CTGACAACAGTGTGAACGAC |
| Maspin Reverse | CAAGCCTTGGGATCAATCATCT |
| Vimentin Forward | GACAATGCGTCTCTGGCACGTCTT |
| Vimentin Reverse | TCCTCCGCCTCCTGCAGGTTCTT |
| p21 Forward | TTTCTCTCGGCTCCCCATGT |
| p21 Reverse | GCTGTATATTCAGCATTGTG |
| Bax Forward | CAGCTCTGAGCAGATCATGAAGACA |
| Bax Reverse | GCCCATCTTCTTCCAGATGGTGAGC |
| MMP2 Forward | TGATGGCATCGCTCAGATCC |
| MMP2 Reverse | GGCCTCGTATACCGCATCAA |
| U6 Forward | GCTTCGGCAGCACATATACTAAAAT |
| U6 Reverse | CGCTTCACGAATTTGCGTGTCAT |

| Supplementary table 2. Demographic data for patients with liver cancer. | | |
| --- | --- | --- |
|  | Men (n = 60) | Women (n = 12) |
| Median Age (years) | 49.0 | 59.4 |
| HBsAg Positive |  |  |
| Yes | 54 | 10 |
| No | 0 | 0 |
| NA | 6 | 2 |
| Grade |  |  |
| High or median | 55 | 10 |
| Low | 0 | 1 |
| NA | 5 | 1 |
| Diameter |  |  |
| ≥ 5 cm | 24 | 5 |
| < 5 cm | 27 | 5 |
| NA | 9 | 2 |
| Number of tumors |  |  |
| Single | 47 | 10 |
| Multiple | 8 | 0 |
| NA | 5 | 2 |
| AFP |  |  |
| ≥ 20 ng/mL | 38 | 6 |
| < 20 ng/mL | 22 | 6 |
| Cirrhosis |  |  |
| Yes | 36 | 8 |
| No | 9 | 1 |
| NA | 15 | 3 |
| BCLC stage |  |  |
| A | 44 | 10 |
| B or C | 11 | 0 |
| NA | 5 | 2 |
| NA, not available | | |

|  | TLNC1 | | |
| --- | --- | --- | --- |
|  | Low (n = 36) | High (n = 36) | *P* |
| Age (years) | 52.0 | 49.4 | 0.421 |
| Sex |  |  | 0.527 |
| Male | 31 | 29 |  |
| Female | 5 | 7 |  |
| Grade |  |  | 0.299 |
| High or median | 34 | 31 |  |
| Low | 0 | 1 |  |
| NA | 2 | 4 |  |
| Diameter |  |  | 0.517 |
| ≥ 5 cm | 16 | 13 |  |
| < 5 cm  NA | 15  5 | 17  6 |  |
| Number of tumors |  |  | 0.423 |
| Single | 30 | 27 |  |
| Multiple  NA | 3  3 | 5  4 |  |
| AFP |  |  | 1.000 |
| ≥ 20 ng/mL | 22 | 22 |  |
| < 20 ng/mL | 14 | 14 |  |
| Cirrhosis |  |  | **0.026** |
| Yes | 18 | 26 |  |
| No  NA | 8  10 | 2  8 |  |
| BCLC stage |  |  | 0.294 |
| A | 29 | 25 |  |
| B or C  NA | 4  3 | 7  4 |  |
| NA, not available | | | |

Supplementary table 3. Correlation between clinicopathologic features in liver cancer patients and expression of TLNC1.

| Supplementary Table 4. Univariable analysis of overall survival and disease-free survival. | | | | | |
| --- | --- | --- | --- | --- | --- |
|  | Overall survival | |  | Disease free survival | |
|  | No. of  cases/total | Hazard Ratio |  | No. of  cases/total | Hazard Ratio |
| Sex |  |  |  |  |  |
| Male | 18/60 | 1.00 (Reference) |  | 36/60 | 1.00 (Reference) |
| Female | 2/12 | 0.44 (0.10-1.91) |  | 9/12 | 0.50 (0.20-1.26) |
| Diameter |  |  |  |  |  |
| ≥ 5 cm | 7/29 | 1.00 (Reference) |  | 16/29 | 1.00 (Reference) |
| < 5 cm | 10/32 | 1.32 (0.50-3.48) |  | 21/32 | 1.42 (0.76-2.67) |
| Number of tumors |  |  |  |  |  |
| Single | 16/57 | 1.00 (Reference) |  | 34/57 | 1.00 (Reference) |
| Multiple | 2/8 | 0.84 (0.19-3.67) |  | 6/8 | 1.34 (0.59-3.03) |
| AFP |  |  |  |  |  |
| ≥ 20 ng/mL  < 20 ng/mL | 13/44  7/28 | 1.00 (Reference)  0.71 (0.28-1.79) |  | 27/44  18/28 | 1.00 (Reference)  0.93 (0.51-1.70) |
| Cirrhosis |  |  |  |  |  |
| Yes | 12/44 | 1.00 (Reference) |  | 26/44 | 1.00 (Reference) |
| No | 1/10 | 0.29 (0.04-2.23) |  | 7/10 | 0.84 (0.34-2.04) |
| BCLC stage |  |  |  |  |  |
| A | 14/54 | 1.00 (Reference) |  | 32/54 | 1.00 (Reference) |
| B or C | 4/11 | 1.66 (0.54-5.09) |  | 8/11 | 1.12 (0.53-2.38) |
| TLNC1 |  |  |  |  |  |
| Low | 8/36 | 1.00 (Reference) |  | 23/36 | 1.00 (Reference) |
| High | 12/36 | 2.28 (0.92-5.63) |  | 22/36 | 1.06 (0.59-1.93) |
